# Influenza A(H5N1) Virus Surveillance at Live Poultry Markets, Cambodia, 2011

**DOI:** 10.3201/eid1902.121201

**Published:** 2013-02

**Authors:** Srey Viseth Horm, San Sorn, Lotfi Allal, Philippe Buchy

**Affiliations:** Author affiliations: Institut Pasteur, Phnom Penh, Cambodia (S.V. Horm, S. Sorn, P. Buchy);; Ministry of Agriculture, Phnom Penh (S. Sorn);; Food and Agriculture Organization, Phnom Penh (L. Allal)

**Keywords:** avian influenza, influenza A(H5N1), subtype H5N1, highly pathogenic avian influenza, influenza, viruses, environment, market, wet markets, live animal markets, live poultry markets, Cambodia, poultry, ducks, birds, surveillance, poultry workers, human avian influenza infection, HPAI, surveillance, transmission, zoonoses

## Abstract

In Cambodia, influenza A(H5N1) virus surveillance at live poultry markets (LPMs) relies on virus isolation from poultry specimens; however, virus is rarely detected by this method. We tested 502 environmental LPM samples: 90 were positive by PCR, 10 by virus isolation. Virus circulation could be better monitored by environmental sampling of LPMs.

Highly pathogenic avian influenza (HPAI) A(H5N1) virus was first detected in 2004 in Cambodia. Since then, outbreaks of subtype H5N1 infection among poultry have been regularly detected, and 21 human cases associated with 19 deaths have been recorded (*1*). 

In Cambodia, where poultry vaccination is not allowed, illegal poultry trade has been a repeated source of reintroduction of the virus (*2*–*4*). Surveillance for subtype H5N1 virus at live poultry markets (LPMs) has been conducted in Cambodia by inoculating cloacal or throat swab specimens from poultry into embryonated chicken eggs; however, virus has rarely been detected by this method (S. Sorn, unpub. data). After outbreaks of subtype H5N1 virus in poultry, the viral genome can be detected for >1 week in environmental samples from the outbreak area; thus, environmental surfaces are potential sources of virus transmission to humans and animals (*5*,*6*). LPMs have also been reported as sources of virus involved in human subtype H5N1 infection (*7*–*9*). 

Birds sold at LPMs can originate from many regions of a country; thus, conducting surveillance for subtype H5N1 virus at these markets would probably be an effective way to monitor the circulation of virus within a country (*7*,*9*,*10*). It has been recommended that samples of drinking water shared by birds maintained within the same cage and samples of poultry feces be used to detect virus within LPMs (*10*,*11*). We performed this study to determine whether subtype H5N1 virus circulation in Cambodia could be better monitored by using environmental sampling in LPMs.

## The Study

**Figure 1:**
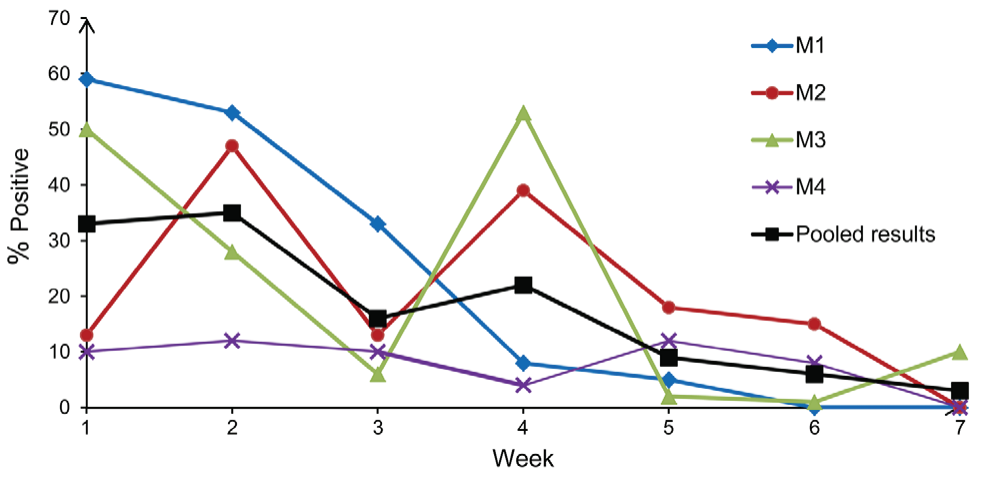
Prevalence of influenza A(H5N1) virus–positive environmental samples from live poultry markets, by collection week, during the Khmer New Year festival, Cambodia, 2011. The New Year festival occurred during week 4 of the study. M1, Orussey market (Phnom Penh); M2, Chamkar Doung market (Phnom Penh); M3, Takeo market (Takeo Province); M4, Kampong Cham market (Kampong Cham Province). Samples positive for the matrix, hemagglutinin 5, and neuraminidase 1 genes by quantitative real-time reverse transcription PCR were considered positive for subtype H5N1 virus. In rare instances, neuraminidase 1–negative samples that were positive for the matrix and hemagglutinin genes were considered positive for subtype H5N1 virus.

In 2011 in Cambodia, environmental samples were collected from 4 LPMs each week for 7 weeks, including during the Khmer New Year festival (Figure 1). Two of the markets were in Phnom Penh, the capital city: Orussey market (M1) and Chamkar Doung market (M2), which also served as an overnight resting place and a place to keep unsold birds from various markets. The third market (M3) was in Takeo (Takeo Province), and the fourth (M4) was in Kampong Cham (Kampong Cham Province). Local chickens, Sampov (domesticated mallards), and Kaki Campbell ducks (domesticated Muscovy ducks) were the only live poultry observed in the markets. Other poultry species were usually available only upon customer request, or they were sold dead.

During the study, we observed that chickens and ducks were mixed together in cages or stalls. In each market, we collected environmental samples from 4–5 poultry cages or from stalls where poultry were gathered. From each sampling site, we collected the following into sterile 50-mL tubes: 50 mL of water used by poultry for drinking, 50 mL of water used to wash carcasses or found on the floor near the slaughtering area, 40–50 g of soil/mud, and 2–3 g of fresh feces present on the soil. Feathers (10–20 g) dropped by birds were gathered and placed in sterile plastic bags.

Virus in water, soil, and mud samples was concentrated, as described (*6*,*12*,*13*), in the biosafety level 3 laboratory at Institut Pasteur, Phnom Penh. Samples were then tested by quantitative real-time reverse transcription PCR (qRT-PCR) targeting the matrix (M), hemagglutinin 5 (H5), and neuraminidase 1 (N1) genes. Feces and feather samples were homogenized with sterile phosphate-buffered saline before nucleic acid extraction and testing. Samples were considered subtype H5N1 virus–positive if qRT-PCR was positive for the M, H5, and N1 genes. The N1 qRT-PCR we used is less sensitive than those used for detection of the M and H5 genes. Thus, in rare instances, we considered N1-negative but H5 and M gene–positive samples to be positive for subtype H5N1 virus. All positive samples were subsequently inoculated into specific pathogen–free embryonated hen eggs for virus isolation (*6*).

Of 502 samples collected, 90 (18%) were positive for subtype H5N1 virus by qRT-PCR, and 10 (2%) were positive by virus isolation (Table). We did not detect >1 positive sample at a time in each cage sampled; thus, each positive sample corresponded to 1 contaminated sampling site. No correlation was observed between viral load measured by qRT-PCR and the ability to isolate the virus in eggs. The overall positivity rate for detection of RNA was >20% for water, feather, and soil/mud samples; the rate was significantly lower for feces samples (6%; p<0.05) (Table). The virus was isolated from 8 (6%) water samples and 2 (2%) soil/mud samples.

**Table:** Results of laboratory testing for influenza A(H5N1) virus in environmental samples from live poultry markets, Cambodia, 2011*

Sample type, market	Samples tested by qRT-PCR			Samples tested by virus isolation†	
	No. positive/no. tested (%)	Total no. positive/total no. tested (%)		No. positive/no. tested (%)	Total no. positive/total no. tested (*%*)
Water		30/145 (21)‡			8/145 (6)§
M1	7/46 (15)			2/46 (4)	
M2	9/37 (24)			3/37 (8)	
M3	11/21 (52)			3/21 (14)	
M4	3/41 (7)			0/41 (0)	
Soil or mud		27/120 (23)‡			2/120 (2)
M1	7/28 (25)			0/28 (0)	
M2	8/28 (29)			1/28 (4)	
M3	9/33 (27)			0/33 (0)	
M4	3/31 (10)			1/31 (3)	
Feces		7/117 (6)‡			0/117 (0)§
M1	3/30 (10)			0/30 (0)	
M2	0/25 (0)			0/25 (0)	
M3	3/33 (9)			0/33 (0)	
M4	1/29 (3)			0/29 (0)	
Feathers		26/120 (22)‡			0/120 (0)§
M1	11/30 (37)			0/30 (0)	
M2	8/28 (29)			0/28 (0)	
M3	4/32 (13)			0/32 (0)	
M4	3/30 (10)			0/30 (0)	
Total		90/502 (18)			10/502 (2)
M1	28/134 (21)¶			2/134 (1)	
M2	25/118 (21)¶			4/118 (3)	
M3	27/119 (23)¶			3/119 (3)	
M4	10/131 (8)¶			1/131 (1)	

*qRT-PCR, quantitative real-time reverse transcription PCR; M1, Orussey market in the capital city of Phnom Penh; M2, Chamkar Doung market in Phnom Penh; M3, a market in Takeo, Takeo Province; M4, a market in Kampong Cham, Kampong Cham Province. †Environmental samples were inoculated into specific pathogen–free embryonated hen eggs for virus isolation. ‡The percentage of samples that were positive was significantly different (by χ^2^ test) for feces vs. water (p = 0.0007), feces vs. soil/mud (p = 0.0003), and feces vs. feathers (p = 0.0005). §The percentage of samples that were positive was significantly different (by χ^2^ test) for water vs. feces (p = 0.001) and water vs. feathers (p = 0.009). ¶The percentage of environmental samples that were positive was significantly different (by χ^2^ test) for M4 vs. M1 (p = 0.002), M4 vs. M2 (p = 0.002), and M4 vs. M3 (p = 0.0008).

Compared with the market in Kampong Cham (M4), the markets in Phnom Penh and Takeo (M1–3) had a higher percentage of samples positive for H5N1virus (8% vs. >20%; p<0.05). Overall, the level of environmental viral contamination in LPMs was highest at the beginning of the study (i.e., 4 weeks before the Khmer New Year, when poultry sales began for the annual festival) and corresponded with intense movement of poultry within the country and higher densities of poultry populations on farms (*2*,*4*); contamination levels tended to progressively decrease, reaching low levels 2 weeks after the event (Figure 1).

**Figure 2:**
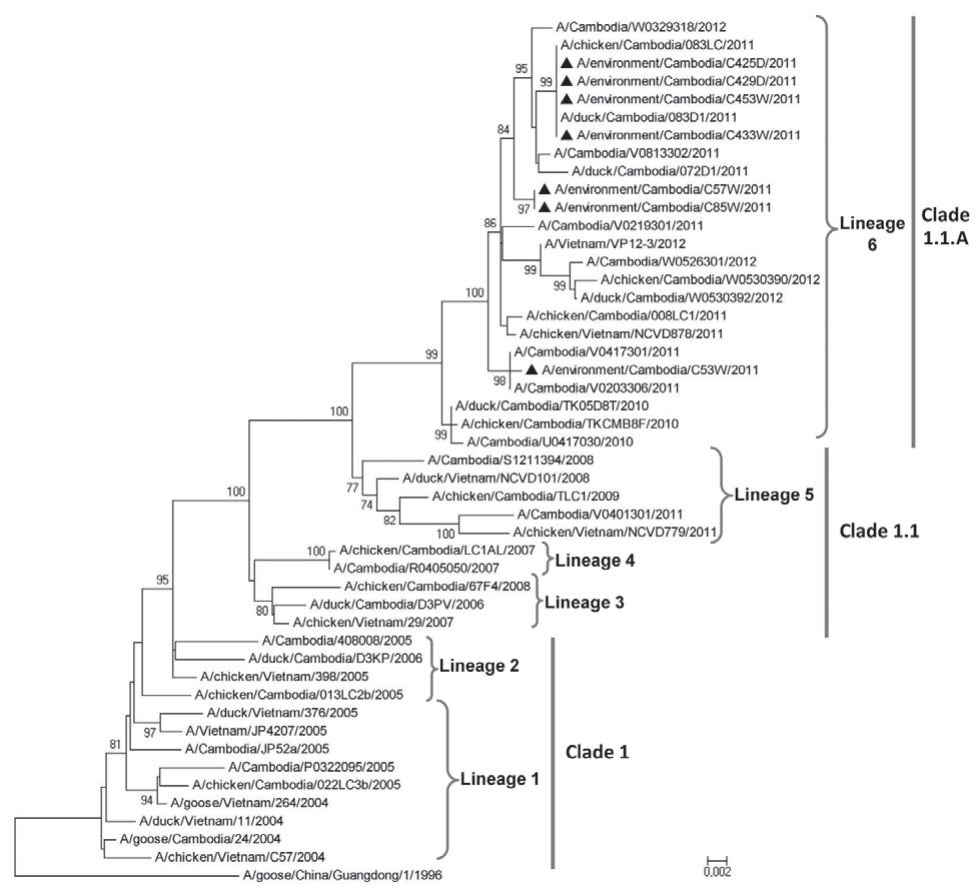
Phylogenetic relationship of the hemagglutinin (HA) gene among various influenza A(H5N1) strains; HA sequences for 48 strains (36 from Cambodia, 11 from Vietnam and one from China) were included in the analysis. Black triangles indicate viruses detected during this study of environmental samples from live poultry markets in Cambodia. Phylogenetic trees were generated by using the distance method and applying the neighbor-joining algorithm with bootstrap analysis (1,000 replicates). Analysis was based on nt 1–1,661 of the HA gene. The trees were rooted to A/goose/China/Guangdong/1/96 (H5N1). Numbers above and below branch nodes indicate bootstrap value of >70%. Scale bar represents the number of nucleotide changes per site. Lineage numbers 1–6, clades, and subclades indicate strains that are grouped in closely related phylogenetic lineages, as described (*14*). All sequences included in the analysis are available in GenBank.

The full genomic sequence of 4 strains and the hemagglutinin sequence of 3 other isolates were generated (GenBank accession nos. JQ673600–JQ673606). Phylogenetic analyses showed that the virus strains detected during this study belong to lineage 6, a group of viruses that seems to be endemic to Cambodia (*14*) (Figure 2), and not to lineage 5, a more regional group of viruses that was also circulating in Cambodia at that time and that includes strains originating from Cambodia and Vietnam. Sequence analyses did not detect reassortment events or mutations associated with higher virulence or increased transmission to humans. Sequences for a virus detected in March 2011 in M3 (Takeo) clustered with sequences for strains isolated from 2 subtype H5N1 virus–infected humans in February near Phnom Penh and in April in Prey Veng Province, respectively. This finding suggests that the strain detected in Takeo was part of a phylogroup that circulated in different regions of the country for several months. Another strain was isolated from M3 on the same day in March. Sequences for that strain did not cluster with those for the isolates from humans in Phnom Penh and Prey Veng Province; however, the strain shared a high degree of homology with a strain detected a week later in Phnom Penh, suggesting the cocirculation in markets of strains with different origins.

## Conclusions

HPAI A(H5N1) virus circulation in Cambodia has traditionally been monitored by using the moderately sensitive egg inoculation method to test cloacal and tracheal swab samples from birds randomly selected from LPMs or farms. Our results show that a more effective approach—especially before and during the main annual festivals, when the movement of poultry within the country is increased—would be to use highly sensitive qRT-PCR to test environmental samples from LPMs. In our study, water samples proved to be the best choice for isolation of infectious subtype H5N1 virus (Table). The lower detection rate of virus among feces samples was expected because the analysis of such samples represents viral shedding by only 1 or a few birds. Available sequence data from other surveillance efforts indicate that all strains detected in this study originated in Cambodia.

In Cambodia, birds not sold the same day they arrive at a live poultry market are transported to an overnight resting place, which is sometimes another market (*7*). Such movement of poultry could increase exposure to environmental contamination with HPAI A(H5N1) virus and thus contribute to virus spread among poultry. It is not known what effect the high levels of HPAI A(H5N1) virus contamination in LPMs in Cambodia have on human health; the effect should be evaluated by conducting clinical and serologic surveillance of vendors and poultry workers.
